# Blood flow stagnation after treatment of a giant internal carotid artery aneurysm: a computed fluid dynamics analysis

**DOI:** 10.1038/s41598-022-11321-6

**Published:** 2022-05-04

**Authors:** Shinsuke Muraoka, Reiya Takagi, Yoshio Araki, Kenji Uda, Masaki Sumitomo, Sho Okamoto, Masahiro Nishihori, Takashi Izumi, Masanori Nakamura, Ryuta Saito

**Affiliations:** 1 Department of Neurosurgery, Kariya Toyota General Hospital, Sumiyoshi Cho 5-15, Kariya, Aichi Japan; 2 Department of Mechanical Engineering, Nagoya Institute of Technology, Gokiso Cho, Showa-ku, Nagoya, Aichi Japan; 3 Department of Neurosurgery, Nagoya University Graduate School of Medicine, Tsurumai Cho 65, Showa-ku, Nagoya, Aichi Japan; 4 Department of Neurosurgery, Toyota Kosei Hospital, Ibobara 500-1, Josui Cho, Toyota, Aichi Japan; 5 Department of Neurosurgery, Aichi Rehabilitation Hospital, Nishigara 1-1, Ebara Cho, Nishio, Aichi Japan

**Keywords:** Translational research, Cerebrovascular disorders, Neurovascular disorders, Stroke

## Abstract

Balloon test occlusion (BTO) is an angiographic test to evaluate ischemic tolerance after permanent occlusion of an internal carotid artery (ICA). BTO can simulate ischemia caused by parent artery occlusion and can be used to select a suitable bypass surgery using specific criteria. On the other hand, a postoperative thrombus can form despite proper case selection, optimal radiological evaluation, and an appropriate surgical strategy. Postoperative ischemic complications related to perforating branches are clinically significant. This simulation study aimed to analyze postoperative flow characteristics and elucidate the cause of ischemic complications related to the perforating branch using computational fluid dynamics (CFD). An unexpected postoperative thrombus formation related to the perforating branch occurred after treating a giant aneurysm in the cavernous portion of the ICA in a patient. Three-dimensional digital subtraction angiography was used to acquire flow data and set up the CFD simulation. The flow simulations were performed at various bypass flow rates. The CFD analysis indicated flow stagnation in the ICA only when surgical treatment using a low-flow bypass graft was performed. Thrombus formation may lead to ischemic complications related to the perforating branch, such as the anterior choroidal artery. BTO did not reflect the influence of bypass blood flow. Therefore, recognizing that blood flow stagnation may occur and comprehensively deciding on the surgical strategy by CFD analysis can be helpful to prevent ischemic complications in patients with giant aneurysms.

## Introduction

Treatment for large or giant aneurysms in the cavernous portion of the internal carotid artery (ICA) remains challenging. Although recent advances in endovascular therapy could provide less invasive treatment for intracranial aneurysms, they do not always offer good outcomes, especially for large or giant ICA aneurysms. Parent artery occlusion (PAO) can lead to intra-aneurysmal thrombosis. However, PAO may occasionally require reconstructive bypass surgery, such as low-flow bypass with a superficial temporal artery (STA)–middle cerebral artery (MCA) bypass or high-flow bypass with a radial artery/saphenous vein graft. A preoperative balloon test occlusion (BTO) of the parent artery is commonly performed to assess the appropriate bypass surgery for each patient. The BTO can simulate the ischemic condition caused by PAO and facilitate the selection of the bypass surgery method using specific criteria. In our institute, we select each treatment based on BTO results.

A postoperative thrombus can form despite proper case selection, optimal radiological evaluation, and an appropriate surgical strategy. Postoperative ischemic complications related to the perforating branches are clinically significant. We recently experienced unfavorable outcomes, such as an unexpected postoperative thrombus formation related to the perforating branch, after treating a giant aneurysm in the cavernous portion of the ICA.

Computational fluid dynamics (CFD) has been used in clinical practice to obtain precise patient-specific hemodynamic parameters, including 3D-velocity, streamline, and particle tracking. This study aimed to analyze postoperative flow characteristics using CFD and elucidate intracranial thrombus formation mechanisms.

## Results

### Analysis of patient flow data

**Figure 1:**
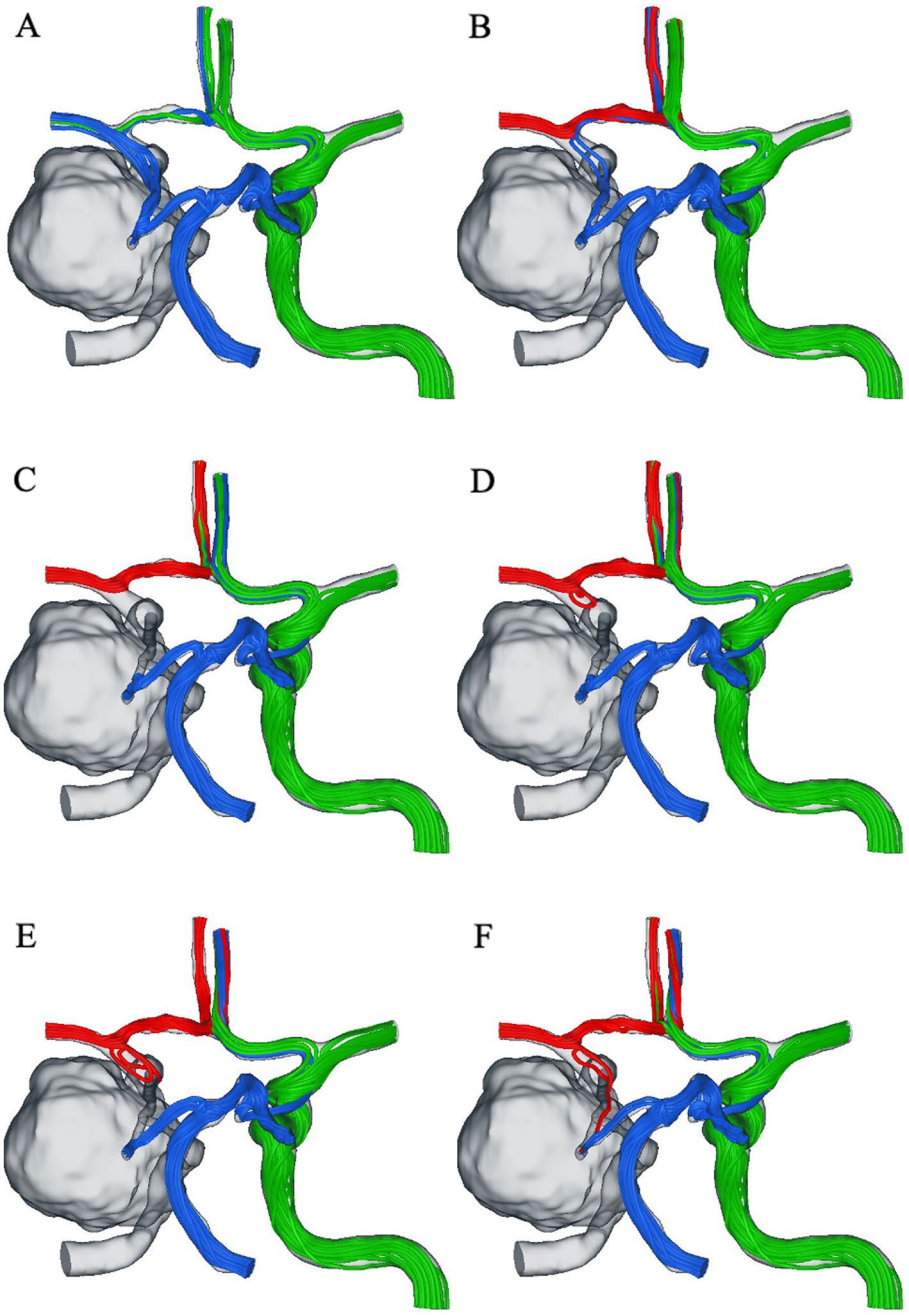
Streamlines are color-coded based on their origin: (red) bypass (right middle cerebral artery, MCA), (blue) BA, and (green) right internal carotid artery. (**a**) right ICA clipped; **(b–f)** bypass to the right MCA. The bypass flow rates were 25 ml/min, 45 ml/min, 50 ml/min, 75, and 100 ml/min.

Figure 1 depicts streamlines color-coded based on their origin. In the posterior–anterior view, the green line indicates blood flow from the left ICA, the red line indicates bypass flow from the STA, and the blue line indicates blood flow from the BA. Without any bypass, the retrograde blood flow of the ICA C8 segment was demonstrated through the posterior communicating artery (Pcom) (Fig. 1a). With a very low bypass flow rate of 25 ml/min, the blood flow of the ICA C8 segment was similar to that in the absence of bypass flows (Fig. 1b). Blood flow stagnation was observed with a low-flow bypass (bypass flow rates = 45 and 50 ml/min) (Fig. 1c,d). With high-flow bypass (bypass flow rates = 75 and 100 ml/min), the anterograde blood flow of the ICA C8 segment was observed (Fig. 1e,f). Consequently, no streamlines were observed in the ICA C8 segment when the bypass flow rate was approximately 50 ml/min, indicating that the flow was stagnant in this segment.

**Figure 2:**
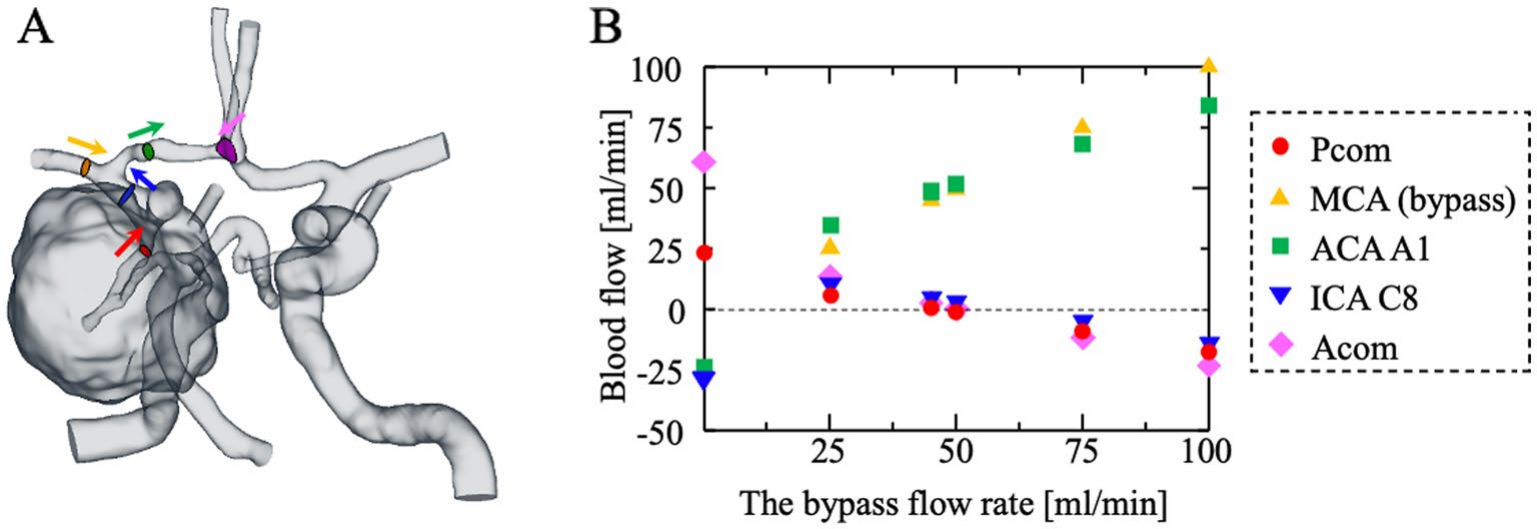
**(a)** The definition of cross-sections where blood flow was obtained. **(b)** Plot of the blood flows in various blood vessels against the bypass flow rates.

The quantitative blood flow analysis is illustrated in Fig. 2. When the bypass flow rate was approximately 50 ml/min, the blood flow was almost zero in the ICA C8 segment, Acom, and Pcom.

**Figure 3:**
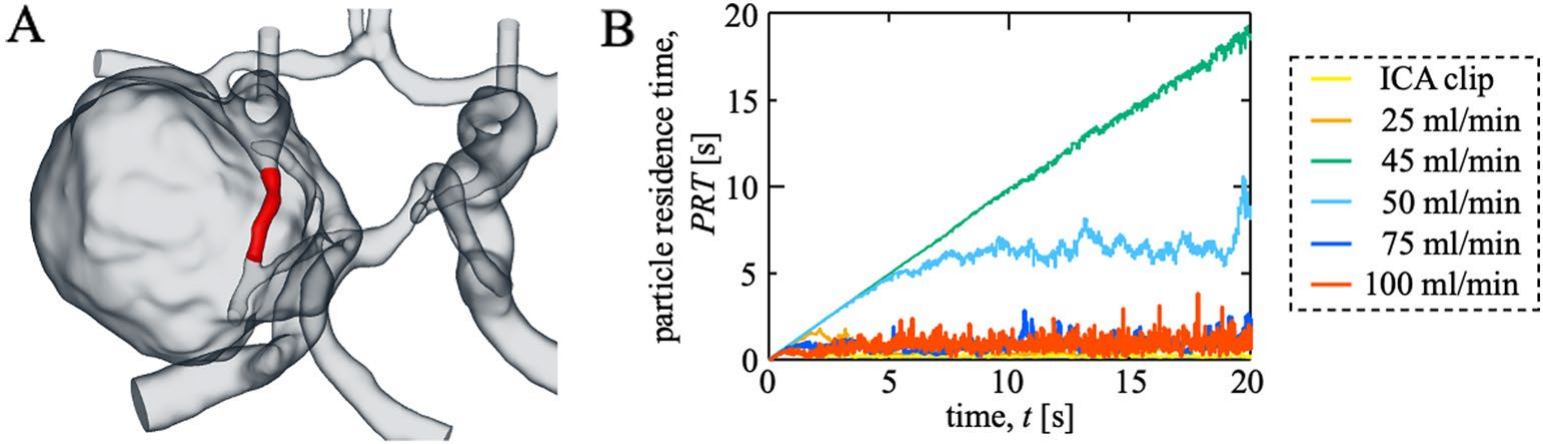
**(a)** Definition of the volume domain, where the mean particle residence time (PRT) was obtained. **(b)** Time variations of PRT for the right internal carotid artery clipping and bypass to the right middle cerebral artery with various flow rates.

Figure 3a depicts the mean PRT for a specific area. The mean PRT was stable and small, except when the bypass flow rate was approximately 50 ml/min (Fig. 3b), indicating that the particles washed out smoothly and had minimal flow stagnation. By contrast, the PRT increased gradually when the bypass flow rate was approximately 50 ml/min, indicating that the risk of thrombus formation increased. Online resources 1–6 reveal the still particle tracking images. The particles were color-coded according to PRT, and those with a large PRT were observed in the case of a bypass flow rate of approximately 50 ml/min, in which the particles were trapped in stagnation and exhibited to-and-fro motions.

## Discussion

In the CFD analysis using the data of postoperative thrombus formation in a patient with a giant aneurysm in the cavernous portion of the ICA, flow stagnation was observed in the ICA C8 segment only when surgical treatment using a low-flow bypass graft was performed. This flow stagnation could not predict preoperative blood flow examination and may have caused the thrombus formation in our case.

Two types of ischemic complications have been reported after surgery for ICA aneurysms with occlusion. One is related to the occlusion of the perforating branch, such as the anterior choroidal artery. Murakami et al. have reported no significant difference in the risk of ischemic complications related to the perforating branch between PAO and any bypass^1^. Hence, ischemic complications related to the perforating branch may be due to mechanisms other than hemodynamic hypoperfusion. The other factor is related to thromboembolic events as most ischemic complications result from surgical procedures.

BTO alone or other CBF studies have performed standard care in the preoperative assessment of patients who require ICA ligation for ICA aneurysms. The sensitivity of BTO can be increased by combining it with CBF studies such as SPECT, PET, and xenon-enhanced CT and stump pressure measurements^2^. Nevertheless, postoperative ischemic complications may occur in approximately 10–20% of patients who pass BTO with the hypotensive challenge^3–5^.

BTO cannot consider the effect of bypass blood flow after ICA ligation for ICA aneurysm surgery; therefore, it does not faithfully express postoperative hemodynamics. Postoperative cerebral infarction in the anterior choroidal artery region, a perforating branch, may be a false negative in BTO. As for the ischemia mechanism in our case, stagnation due to the bypass blood flow collision may have been the cause. For the treatment with an ICA giant aneurysm, we have to do the preoperative simulation considering the bypass blood flow using some modalities.

Stagnation of blood flow has been reported in cardiovascular surgery. Aortic valve bypass surgery is an alternative to aortic valve replacement in high-risk patients with aortic stenosis. Aortic thrombus is severe and may be caused by blood flow stagnation arising from competition between the antegrade and retrograde flows. Kawahito et al. confirmed flow stagnation in the aorta by CFD analysis^6^.

Blood flow stagnation in cerebrovascular surgery has not been previously reported. PAO with bypass vessels invariably results in blood flow competition at any site. This is due to retrograde blood flow through the bypass vessel and antegrade blood flow from the contralateral internal carotid artery. Although hemodynamics are expected to stabilize during the chronic phase, this blood flow collision will be more critical during the acute phase. Therefore, treatment of large or giant internal carotid artery aneurysms with a flow-diverting stent may make sense as the optimal treatment to preserve the progressive blood flow.

This study has some limitations. First, these results are based only on the anatomical data of a specific patient. Hence, the results may differ depending on each blood vessel's branch angle and blood vessel diameter; therefore, analyzing each case is necessary. Second, compliance with the blood vessel wall was not considered in our CFD model. The assumed rigidity could have affected blood flow dynamics. However, including elasticity as a study variable requires a fluid–structure interaction analysis. The material properties and wall thickness necessary for the structure simulation are difficult to obtain for each patient. Cases with aneurysms have more difficulties in determining them as the material properties, and wall thickness would be spatially heterogeneous at aneurysms. Furthermore, residual stresses within the vascular wall are unknown, raising how to define the blood vessel's natural state (zero-stress state). Mechanical interaction with neighboring brain tissues brings additional difficulties, making solid analysis more challenging. As shown in Torii et al.^7^ the inclusion of elastic walls provides quantitative differences in some fluid mechanical quantities such as wall shear stress near the aneurysms compared with the results obtained with the rigid wall. However, overall flow patterns obtained with the fluid–structure interaction analysis remain the same as those obtained with the rigid wall. The primary aim of the present study is to investigate postoperative flow characteristics and elucidate the cause of ischemic complications related to the perforating branch. CFD with the rigid wall assumption would have provided reasonably convincing results for this aim. Future works will include fluid–structure interaction simulations to confirm the present results, although they may be associated with uncertainties, and numerical complexity increases significantly.

## Conclusion

Postoperative ischemic complications related to the perforating branch are clinically significant in patients with giant aneurysms of the cavernous portion of the ICA. The CFD analysis revealed that flow stagnation occurred only in the ICA C8 segment when surgical treatment using a low-flow bypass graft was performed. Thrombus formation may lead to ischemic complications related to the perforating branch, such as the anterior choroidal artery. Therefore, recognizing that blood flow stagnation may occur and comprehensively deciding on the surgical strategy by CFD analysis can be helpful to prevent ischemic complications in patients with giant aneurysms.

## Methods

This study was approved by the institutional review board of Nagoya University Graduate School of Medicine (approval number: 2016-0437). All procedures performed were in accordance with the ethical standards of the Declaration of Helsinki. All experiments were performed in accordance with CARE guideline. Informed concent was obtained from the patient’s families.

### Study design

This simulation study analyzed blood flow stagnation in the ICA in these case scenarios (bypass blood flow rates of 0, 25, 45, 50, 75, and 100 ml/min) based on a patient's preoperative condition data.

### Operative strategy

**Figure 4:**
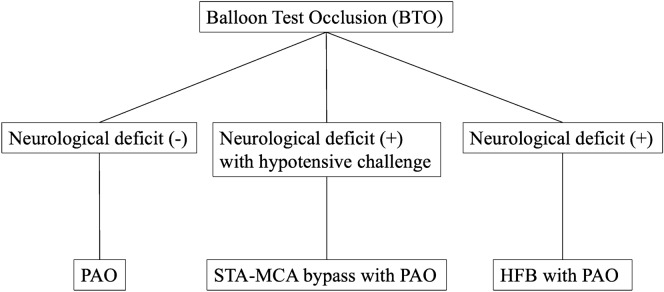
Operative strategy for large or giant aneurysms in the cavernous portion of the internal carotid artery.

Our institute’s policy is essentially based on BTO with a hypotensive challenge (Fig. 4)^8^. In cases in which retrograde dome filling from collateral flow was observed during BTO, a parent artery occlusion (PAO) was conducted. Otherwise, proximal ICA ligation of the cervical portion was performed with no retrograde dome filling from the collateral flow during BTO. Treatment for contralateral CCA was based on the second BTO’s results. A high-flow bypass with PAO was administered if a patient exhibited a neurologic deficit during a 20-min BTO. An STA–MCA bypass with PAO was performed in patients who demonstrated neurologic deficits during a BTO test within a 15-min hypotensive challenge. Without bypass, PAO was performed in patients who did not demonstrate neurologic deficits during the BTO test within 15 min of a hypotensive challenge.

### Digital subtraction angiography

Three-dimensional digital subtraction angiography (3D-DSA) was performed by femoral catheterization using the Seldinger technique with a biplane DSA unit with rotational capabilities (Axiom Artis dTA; Siemens Healthcare, Erlangen, Germany). Typically, 4–10 ml of nonionic contrast medium (Iopamidol, 300 mg of iodine per ml; Teva Takeda Pharma, Nagoya, Japan) was used per acquisition. The spatial resolution was 0.32 × 0.32 mm. Standard anteroposterior and lateral DSA images were obtained with the catheter in the four significant arteries (the common carotid arteries and vertebral arteries). The single 3D rotational angiographic acquisition is typically performed to evaluate aneurysm shape and surrounding blood vessels. Images were reconstructed using a 256 × 256 matrix. Rotational angiographic data were transferred to an independent workstation (Syngo Workplace; Siemens Healthcare, Erlangen, Germany) to generate 3D reformatted images. 3D-DSA data were immediately sent to an adjacent 3D workstation (Siemens Medical Solutions, Erlangen, Germany). An experienced endovascular staff reviewer evaluated the conventional DSA and discussed the operative strategy with the surgical intervention staff.

### The CFD model

Postoperative blood flow characteristics were evaluated by CFD analysis. The vascular geometry was reconstructed from the 3D-DSA images, and the surface was smoothed using a 3D slicer, which is an image-computing platform (ver. 4.10.2, The Slicer Community, Harvard, USA). Five layers of prism meshes were created near the vessel wall, and the remaining geometry was filled with polyhedral meshes. The total number of meshes was approximately 600,000. Blood was treated as an incompressible Newtonian fluid with a density of 1060 kg/m^3^ and viscosity of 0.004 Pa∙s.

Flow simulation in the 3D domain of the vascular geometry obtained from the 3D-DSA images was conducted using scFlow (MSC software, Japan). The flow was assumed steady. A no-slip condition was applied to the wall, and the vessel wall was assumed to be rigid.

Bypass surgery of the left MCA with clipping of the left ICA was simulated by providing inflow to the left MCA. Previous reports have revealed low-flow bypass grafts, such as the STA permit 15–50 ml/min flow velocity^9–11^. High-flow bypass grafts, such as a radial artery or saphenous vein graft, have flow rates of 50–150 ml/min^9,12^. Flow simulations were implemented at various intracranial blood flows of the bypass graft (bypass blood flow of 0, 25, 45, 50, 75, and 100 ml/min). Based on the patient's own DSA data, inflows of 235, 5, and 114 ml/min were administered to the right ICA, basilar artery (BA), and left ophthalmic artery, respectively. These blood flow velocities were obtained from the patients’ data. A 0D blood flow model was assigned to the rest of the blood vessels, numerically coupled with blood flow in a 3D domain.

For comparison, ICA clipping without bypass surgery was also simulated. The boundary condition was the same as in the cases with bypass surgery, except for the left MCA. In this case, the 0D blood flow model was assigned to the left MCA instead of defining the flow rate.

### Particle residence time

The particle residence time (PRT) was calculated for the quantitative evaluation of particle tracking. The trajectories of massless and volumeless particles were calculated using the predictor–corrector method considering the flow velocity fields.

### Patient

**Figure 5:**
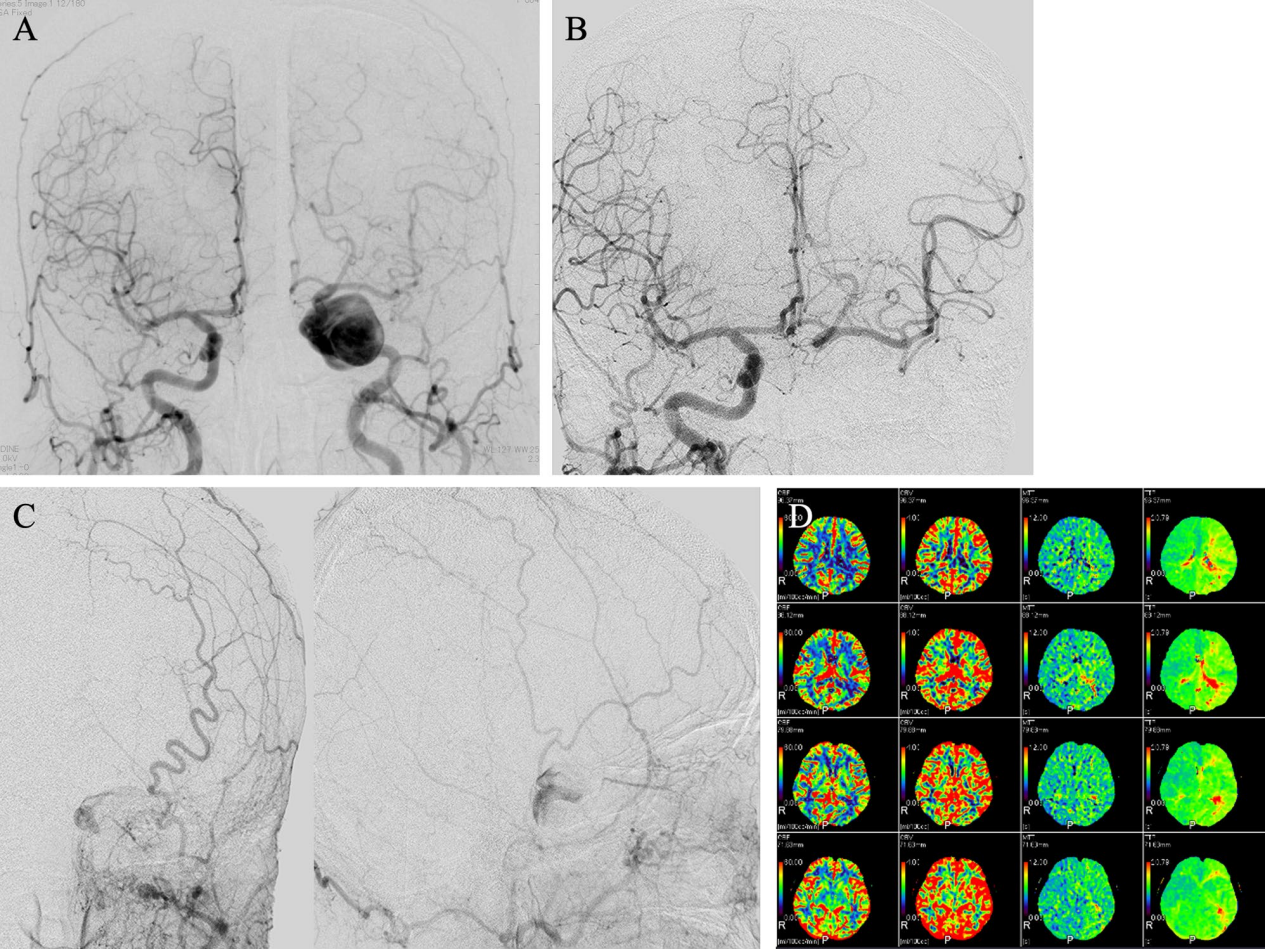
Digital subtraction angiography (DSA) and balloon test occlusion test of the left internal carotid artery (ICA). (**a**) Anterior–posterior (AP) view of the left and right common carotid artery angiograms. (**b**) BTO of the left ICA demonstrating collateral flow through the anterior communicating artery. (**c**) AP and lateral views of concurrent injection of the left external carotid artery showing retrograde flow to the aneurysm via the ophthalmic artery. (**d**) Perfusion computed tomography with BTO. Cerebral blood flow, cerebral blood volume, mean transit time, and time to peak are shown from left to right.

A 64-year-old woman visited an ophthalmologist for diplopia for one month. She was referred to our department for abductor nerve palsy. Brain magnetic resonance imaging revealed a 25-mm left giant ICA cavernous segment aneurysm. A 3D-DSA was performed (Fig. 5); the left common carotid artery angiography (CCAG) suggested a left giant ICA cavernous segment aneurysm. The right CCAG with a left ICA balloon occlusion indicated adequate perfusion of the contralateral MCA territory via the anterior communicating artery (Acom). Left external carotid artery angiography with left ICA balloon occlusion indicated retrograde blood flow to the aneurysm via the left ophthalmic artery. No neurological deficit was observed with left BTO, although aphasia occurred during the BTO test with a hypotensive challenge. Computed tomography (CT) perfusion imaging during the BTO test indicated no change in cerebral blood flow (CBF), a slight increase in cerebral blood volume (CBV), and an extension of mean transit time. This CT perfusion image suggested that peripheral arterial dilation led to preserving the left CBF. Preoperative all general coagulation system tests were within normal limits.

### Surgical procedure

The patient was in supine, and her head was rotated toward the right side at approximately 30°. A left frontotemporal craniotomy was performed followed by cervical ICA surgical ligation and a double STA–MCA bypass surgery. Intraoperative indocyanine green video angiography revealed good bypass flow. Sensory-evoked potential and motor-evoked potential monitorings were normal during the surgery.

### Postoperative course

**Figure 6:**
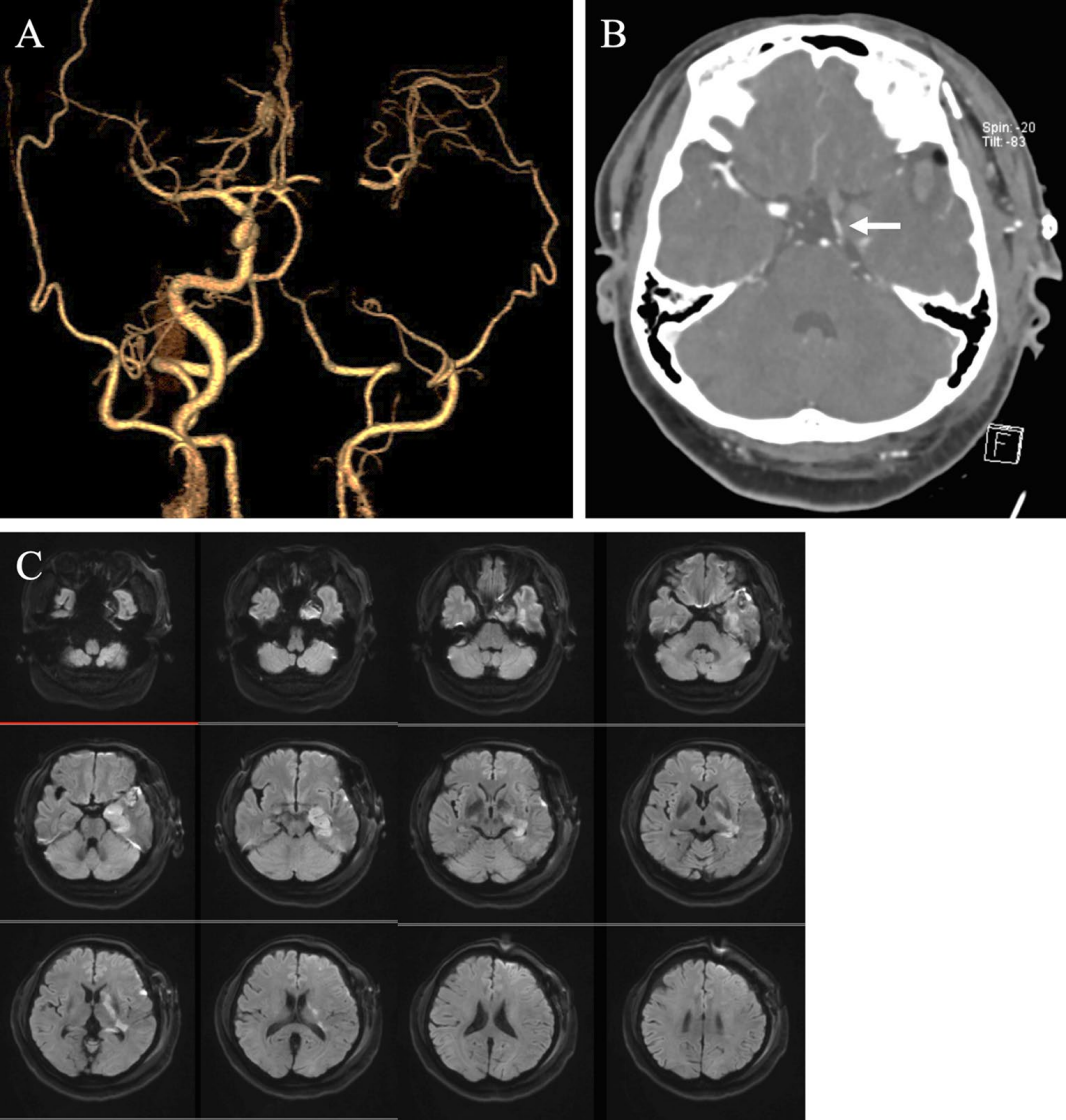
Postoperative computed tomography angiography and magnetic resonance imaging. (**a**) Double bypass flow was observed, although thrombus formation occurred until the left internal carotid artery terminus. (**b**) The left posterior communicating artery flow was confirmed (white arrow). (**c**) Cerebral infarction is observed in the anterior choroidal artery territory.

After the operation, the patient woke up with good consciousness. The patient was transferred to the intensive care unit with no remarkable neurological deficits. Approximately 12 h postoperatively, contralateral hemiplegia, hemianesthesia, and homonymous hemianopsia occurred with occlusion of the anterior choroidal artery. Brain magnetic resonance angiography revealed an acute thrombus formation at the C8 segment of the ICA with good bypass patency (Fig. 6). Despite systemic anticoagulation and antiplatelet therapy, the thrombus formation progressed. On postoperative day 4, the patient died of hemorrhagic cerebral infarction.

### Ethics approval

All procedures performed were in accordance with the ethical standards of the Declaration of Helsinki. This study was approved by the institutional review board of Nagoya University Graduate School of Medicine (approval number: 2016-0437).

### Consent to participate

All experiments were performed in accordance with relevant named guidelines and regulations and informed concent was obtained from the patient’s families.

## Supplementary Information

Supplementary Legends.

Supplementary Video 1.

Supplementary Video 2.

Supplementary Video 3.

Supplementary Video 4.

Supplementary Video 5.

Supplementary Video 6.

## Data Availability

The datasets used and/or analyzed during the current study available from the corresponding author on reasonable request.

## References

[1] Murakami K, Shimizu H, Matsumoto Y, Tominaga T (2009). Acute ischemic complications after therapeutic parent artery occlusion with revascularization for complex internal carotid artery aneurysms. Surg. Neurol..

[2] Sudhakar KV, Sawlani V, Phadke RV, Kumar S, Ahmed S, Gujral RB (2000). Temporary balloon occlusion of internal carotid artery: A simple and reliable clinical test. Neurol. India..

[3] Sekhar LN, Patel SJ (1993). Permanent occlusion of the internal carotid artery during skull-base and vascular surgery: Is it safe?. Am. J. Otol..

[4] Segal DH, Sen C, Bederson JB, Catalano P, Sacher M, Stollman AL (1995). Predictive value of balloon test occlusion of the internal carotid artery. Skull Base Surg..

[5] Dare AO, Chaloupka JC, Putman CM, Fayad PB, Awad IA (1998). Failure of the hypotensive provocative test during temporary balloon test occlusion of the internal carotid artery to predict delayed hemodynamic ischemia after therapeutic carotid occlusion. Surg. Neurol..

[6] Kawahito K, Kimura N, Komiya K, Nakamura M, Misawa Y (2017). Blood flow competition after aortic valve bypass: An evaluation using computational fluid dynamics. Interact. Cardiovasc. Thorac. Surg..

[7] Torii R, Oshima M, Kobayashi T, Takagi K, Tezduyar TE (2006). Fluid–structure interaction modeling of aneurysmal conditions with high and normal blood pressures. Comput. Mech..

[8] Uozumi Y, Okamoto S, Araki Y, Izumi T, Matsubara N, Yokoyama K (2015). Treatment of symptomatic bilateral cavernous carotid aneurysms: long-term results of 6 cases. J. Stroke Cerebrovasc. Dis..

[9] Baaj AA, Agazzi S, van Loveren H (2009). Graft selection in cerebral revascularization. Neurosurg. Focus..

[10] Alaraj A, Ashley WW, Charbel FT, Amin-Hanjani S (2008). The superficial temporal artery trunk as a donor vessel in cerebral revascularization: Benefits and pitfalls. Neurosurg. Focus..

[11] Kim JY, Jo KW, Kim YW, Kim SR, Park IS, Baik MW (2010). Changes in bypass flow during temporary occlusion of unused branch of superficial temporal artery. J. Korean Neurosurg. Soc..

[12] Kocaeli H, Andaluz N, Choutka O, Zuccarello M (2008). Use of radial artery grafts in extracranial-intracranial revascularization procedures. Neurosurg. Focus..

