# Supplementary material for: Blood flow stagnation after treatment of a giant internal carotid artery aneurysm: a computed fluid dynamics analysis
Source: Sci Rep. 2022 May 4;12:7283. doi: 10.1038/s41598-022-11321-6 (PMC9068907; doi:10.1038/s41598-022-11321-6)
Supplement: Supplementary file 1 — Supplementary Legends. [file 41598_2022_11321_MOESM1_ESM.docx]

**SUPLEMENTARY VIDEO LEGENDS**

**Video 1**

Postoperative blood flow characteristics (bypass flow rate=0ml/min) using PRT. This situation was imitated parent artery occlusion without any bypass vessels. Under this situation, there was no stagnation around the left ICA C8 segment.

**Video 2**

Postoperative blood flow characteristics (bypass flow rate=25 ml/min) using PRT. This situation imitated parent artery occlusion with very low-flow bypass vessels. Under this situation, stagnation was slight around the left ICA C8 segment.

**Video 3**

Postoperative blood flow characteristics (bypass flow rate=45 ml/min) using PRT. This situation imitated parent artery occlusion with low-flow bypass vessels, such as the superficial temporal artery. Under this situation, stagnation was present around the left ICA C8 segment.

**Video 4**

Postoperative blood flow characteristics (bypass flow rate=50 ml/min) using PRT. This situation imitated parent artery occlusion with low-flow bypass vessels, such as the superficial temporal artery. Under this situation, stagnation was present around the left ICA C8 segment.

**Video 5**

Postoperative blood flow characteristics (bypass flow rate=75 ml/min) using PRT. This situation imitated parent artery occlusion with high-flow bypass vessels, such as the radial artery. Under this situation, no stagnation was observed around the left ICA C8 segment.

**Video 6**

Postoperative blood flow characteristics (bypass flow rate=100 ml/min) using PRT. This situation imitated parent artery occlusion with high-low flow bypass vessels, such as the saphenous vein. Under this situation, no stagnation was observed around the left ICA C8 segment.
